# Cell-Type Annotation for scATAC-Seq Data by Integrating Chromatin Accessibility and Genome Sequence

**DOI:** 10.3390/biom15070938

**Published:** 2025-06-27

**Authors:** Guo Wei, Long Wang, Yan Liu, Xiaohui Zhang

**Affiliations:** 1 State Key Laboratory of Pharmaceutical Biotechnology, School of Life Sciences, Nanjing University, Nanjing 210000, China; wanglong@nju.edu.cn; 2 Department of Computer Science, Yangzhou University, Yangzhou 225100, China; 008474@yzu.edu.cn

**Keywords:** graph attention networks, convolutional neural networks, cross-omics, genome

## Abstract

Single-cell Assay for Transposase-Accessible Chromatin using sequencing (scATAC-seq) technology enables single-cell resolution analysis of chromatin accessibility, offering critical insights into gene regulation, epigenetic heterogeneity, and cellular differentiation across various biological contexts. However, existing cell annotation methods face notable limitations. Cross-omics approaches, which rely on single-cell RNA sequencing (scRNA-seq) as a reference, often struggle with data alignment due to fundamental differences between transcriptional and chromatin accessibility modalities. Meanwhile, intra-omics methods, which rely solely on scATAC-seq data, are frequently affected by batch effects and fail to fully utilize genomic sequence information for accurate annotation. To address these challenges, we propose scAttG, a novel deep learning framework that integrates graph attention networks (GATs) and convolutional neural networks (CNNs) to capture both chromatin accessibility signals and genomic sequence features. By utilizing the nucleotide sequences corresponding to scATAC-seq peaks, scAttG enhances both the robustness and accuracy of cell-type annotation. Experimental results across multiple scATAC-seq datasets suggest that scAttG generally performs favorably compared to existing methods, showing competitive performance in single-cell chromatin accessibility-based cell-type annotation.

## 1. Introduction

scATAC-seq technology enables the characterization of chromatin accessibility at single-cell resolution, facilitating the analysis of gene regulatory networks [1] and epigenetic heterogeneity  [2]. This technique is widely applied in studies of cell differentiation, disease mechanisms, and the exploration of immune and tumor microenvironments [3,4]. However, due to the high dimensionality, extreme sparsity, and significant technical noise in scATAC-seq data, accurate cell-type annotation remains a major challenge.

Current scATAC-seq cell annotation methods primarily depend on reference datasets with pre-annotated cell types. These methods employ machine learning models to capture the relationships between chromatin accessibility patterns and cell types from a reference dataset, subsequently applying this knowledge to annotate the query dataset [5,6]. Based on the nature of the reference data, these annotation approaches can be classified into intra-omics and cross-omics methods.

Intra-omics methods utilize well-annotated scATAC-seq datasets as references, leveraging chromatin accessibility signals to map cell types onto unannotated data. For instance, Cellcano [7] employs a two-stage supervised learning framework for cell-type annotation. In the first stage, a multilayer perceptron (MLP) [8] is trained on the reference dataset and used to predict cell types in the target dataset. Based on the initial predictions, Cellcano selects a subset of confidently predicted target cells, referred to as “anchors”, to construct a new training set. In the second stage, it trains a self-knowledge distillation model using pseudo-labeling on these anchor cells. The trained model is then applied to predict the cell types of the remaining non-anchor cells. scATAnno [9] begins by extracting cell embedding features from the reference dataset using the SnapATAC2 [10] method. It then applies Harmony [11] to correct batch effects between the reference and query datasets. Finally, the k-nearest neighbor (KNN) algorithm [12] is used to assign cell types to the query cells. scEmbed is an unsupervised machine learning framework that first learns the embeddings of genomic regulatory regions from the reference dataset. These embeddings are subsequently used to compute cell embeddings. By leveraging transfer learning, scEmbed [13] transfers the learned representations to the query dataset, enabling accurate cell-type annotation. EpiAnno [14], in contrast, utilizes a Bayesian neural network framework [15] to train a model specifically for cell-type annotation, incorporating probabilistic inference to enhance robustness and accuracy.

Cross-omics methods adopt scRNA-seq as a reference, aligning scATAC-seq and scRNA-seq data within a shared embedding space to facilitate cross-modality cell annotation. However, inherent differences in data structure and modality-specific biases pose substantial challenges to alignment. To mitigate these issues, various strategies have been developed, including manifold alignment-based approaches [16], which preserve the local geometric properties of each dataset, and gene activity matrix (GAM) [17] transformation methods, which infer gene regulatory activity from chromatin accessibility signals.

Representative manifold alignment-based methods for integrating single-cell multi-omics data include MATCHER [18], SCIM [19], MMD-MA [20], and GLUE [21]. MATCHER is a Gaussian process latent variable model that exploits correlations between transcriptomic and epigenetic measurements to reconstruct differentiation trajectories in embryonic stem cells. SCIM and MMD-MA process raw scATAC-seq and scRNA-seq data through separate neural networks, aligning their latent features using either adversarial learning [22] or a maximum mean discrepancy (MMD) minimization loss [23] to bridge the differences between modalities. GLUE integrates prior biological knowledge of feature interactions across modalities to guide a graph neural network (GNN) [24] in learning shared cell embeddings, improving alignment and cell-type annotation across datasets. Representative GAM transformation-based methods for scATAC-seq cell annotation include scJoint [25], scDART [26], AtacAnnoR [27], and scNCL [28]. In scJoint, AtacAnnoR, and scNCL, the GAM is computed from the chromatin accessibility peak matrix obtained from single-cell RNA-ATAC joint sequencing data. After transforming scATAC-seq data into GAM, scJoint applies a semi-supervised transfer learning strategy to learn joint embeddings between scRNA-seq and scATAC-seq, followed by label propagation based on these learned embeddings. scNCL employs contrastive learning [29,30] to preserve the neighborhood structure of the reference scRNA-seq data in an embedding space, thereby enhancing the alignment between scATAC-seq and scRNA-seq datasets. AtacAnnoR follows a two-step annotation process for scATAC-seq data using scRNA-seq as a reference. First, it assigns cell-type labels to a subset of query cells by comparing gene activity profiles derived from scATAC-seq with reference gene expression profiles. Then, it applies a weighted KNN [31] to annotate the remaining query cells. Unlike scJoint and scNCL, scDART utilizes a fully connected neural network to learn a direct mapping between regulatory regions in scATAC-seq data and genes in scRNA-seq data. The trained model is subsequently used to annotate cell types in scATAC-seq data, offering an alternative deep learning-based approach.

Although cross-omics cell-type annotation has achieved notable success, aligning scRNA-seq and scATAC-seq data within a unified latent space remains a considerable challenge. This challenge stems from the fundamental differences between scRNA-seq and scATAC-seq, including variations in feature dimensionality, and the biological nature of the signals—gene expression versus chromatin accessibility, respectively. These differences complicate the integration process and can result in suboptimal annotation performance. At the same time, the increasing availability of high-quality, well-annotated scATAC-seq datasets has highlighted the growing potential of intra-omics scATAC-seq cell-type annotation methods. However, two major challenges remain:

1. Alleviating batch effects [32,33] between the reference and query datasets, including differences introduced by variations in experimental protocols, sequencing depth, sample handling, or platform-specific biases. These batch-induced discrepancies can obscure true biological signals and negatively impact the accuracy and robustness of downstream cell-type annotation.
2. The majority of existing methods primarily focus on peak matrices, often overlooking genomic sequence information [34]. Fully leveraging genomic sequence features, such as nucleotide composition, transcription factor binding motifs, and local sequence patterns extracted from accessible chromatin regions, to enhance cell-type annotation accuracy remains an open research question. Addressing this challenge requires the development of more integrative computational approaches that can effectively incorporate such sequence-derived information alongside chromatin accessibility signals.

In this study, we focus on the intra-omics strategy and propose scAttG, a novel deep learning framework for scATAC-seq cell-type annotation, which integrates a graph attention network (GAT) with convolutional neural networks (CNNs) [35]. scAttG consists of two main components: (1) Genomic Feature Representation Learning: A CNN is utilized to extract feature representations of individual cells from their corresponding genomic sequences. These learned features capture regulatory sequence context, such as shared transcription factor binding motifs or conserved elements within accessible chromatin regions. Based on these representations, neighbor-joining matrices are constructed to model cell–cell relationships, effectively reflecting similarities in regulatory potential and underlying sequence architecture; (2) Graph Attention-Based Aggregation: Using the adjacency matrix derived from the first step, a GAT aggregates chromatin accessibility information from the scATAC-seq peak matrix to refine cell feature representations. This approach enables scAttG to integrate both genomic sequence features and chromatin accessibility signals, improving the accuracy and robustness of scATAC-seq cell type annotation.

## 2. Materials and Methods

### 2.1. The Architecture of scAttG

**Figure 1:**
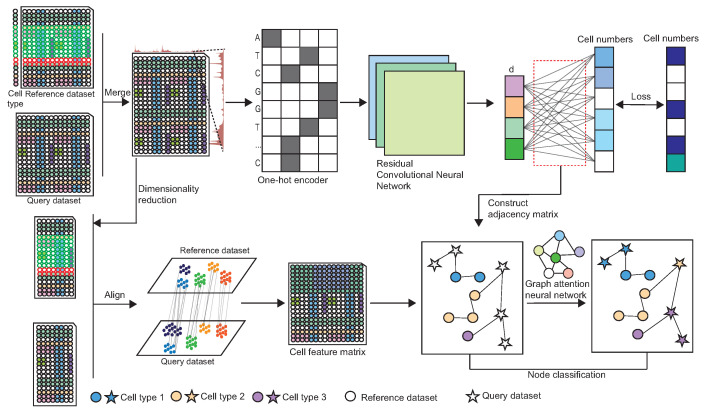
Computational framework of scAttG.

scAttG is a scalable and highly accurate framework that integrates DNA sequence information and chromatin accessibility data to facilitate label propagation across scATAC-seq datasets. The computational workflow of scAttG, as illustrated in Figure 1, consists of two key stages, sequence information extraction and cell-type annotation, incorporating dimensionality reduction, batch effect correction, feature fusion, and cell-type annotation. In the sequence information extraction stage, scAttG employs a CNN [36] to extract low-dimensional representations from DNA sequences associated with chromatin accessibility peaks in both the reference and query datasets. These representations are then used to construct an adjacency matrix, capturing the relationships between cells based on genomic sequence similarity. During cell-type annotation, the chromatin accessibility peak matrix undergoes dimensionality reduction and batch effect correction, generating cell embeddings that capture meaningful biological features. These embeddings, along with the adjacency matrix, are then input into a GAT for node classification [37]. In this process, unknown cells are treated as nodes with unknown labels, while reference dataset cells serve as nodes with known labels. The GAT-based node classification enables label propagation, effectively transferring cell-type annotations from the reference dataset to the query dataset, leading to highly accurate cell-type annotations.

### 2.2. Construct the Adjacency Matrix Based on the Genome Information

#### 2.2.1. DNA Sequence Information Extraction

CNNs are powerful feature extractors widely applied in computer vision  [38], natural language processing (NLP)  [39], and bioinformatics  [40]. Inspired by previous work, this study employs a 1D convolutional neural network (1D-CNN) to process binary accessibility vectors for each chromatin accessibility peak, leveraging the underlying DNA sequence to extract cell-meaningful representations. At the core of the CNN model of scAttG, the input DNA sequences are first transformed using one-hot encoding—a scheme in which each nucleotide (A, C, G, T) is represented as a distinct binary vector (e.g., A = [1, 0, 0, 0], C = [0, 1, 0, 0], etc.). This representation preserves base-level sequence information while allowing the neural network to treat the data numerically. These encoded sequences are then passed through an initial convolutional layer to extract local motif patterns relevant to regulatory activity. This layer applies a 1D convolution (kernel size = 17, output channels = 288), followed by ReLU activation, and max pooling, effectively reducing sequence dimensionality while retaining key feature representations. The feature extraction process is hierarchically structured through a series of ConvTower blocks, each comprising two 1D convolutional layers, a batch normalization layer, and an ECA attention module  [34]. The ECA-Net mechanism adaptively determines cross-channel dependencies based on the number of channels, leveraging global pooling and depthwise convolution to efficiently capture long-range interactions while maintaining computational efficiency. The convolutional outputs are progressively downsampled through max pooling layers, reducing sequence length while enhancing feature richness. Additionally, residual connections with downsampling projections are incorporated to stabilize gradient flow and facilitate robust feature propagation. Once the hierarchical convolutional process is complete, the feature maps are passed through a post-convolutional refinement layer, where a 1×1 convolution is applied to condense information into a lower-dimensional representation while preserving the spatial structure of the feature maps. The output is then flattened into a vector format and fed into a fully connected projection layer (hidden size = 64), which further refines the learned feature embeddings. To improve generalization and mitigate overfitting, a dropout layer (dropout probability = 0.2) is introduced.

#### 2.2.2. Cell Representation Extraction

The learned feature vectors are first processed through a d-unit bottleneck layer (d = 64) to capture low-dimensional embeddings of the accessibility peaks. These compressed representations are then passed into a dense layer, which predicts the peak reachability for N cells. The parameters of the entire network are iteratively optimized using binary cross-entropy loss, ensuring robust peak accessibility predictions. To further refine cell embeddings, the learned weights in the dense network are transposed, producing a 64-dimensional representation for each of the N cells. To alleviate batch effects between reference and query datasets, a parallel dense layer is introduced at the bottleneck layer. This additional network learns batch-specific peak reachability, which is then multiplied by a cell-by-cell batch matrix to estimate the batch-associated peak reachability for each cell. The resulting batch-adjusted vector is then added to the existing peak reachability scores, ensuring that cell annotations remain robust across different batches and experimental conditions.

#### 2.2.3. Training Details of CNN Model

During the training process, the initial learning rate and batch size are set to $1\times 10^{-3}$ and 256, ensuring a stable yet efficient optimization trajectory. To evaluate model performance, a validation set comprising 2000 randomly sampled cells is drawn from the merged dataset—which integrates both the reference and query datasets based on peak accessibility. The training process is configured to run for a maximum of 300 epochs, providing ample opportunity for the model to achieve convergence. The optimal parameters, determined through validation performance, are then used to generate the final embeddings for both the query dataset and the reference dataset, ensuring high-fidelity representations for downstream tasks.

#### 2.2.4. Construct the Adjacency Matrix

To construct the graph structure from cell embeddings generated by the above step, we employ a KNN graph approach, where each cell is treated as a node and connected to its k most similar neighbors based on Euclidean distance in the embedding space. Given a set of cell embeddings $X\in R^{n\times d}$, where *n* is the number of cells and *d* is the feature dimension, we compute pairwise distances and identify the k-nearest neighbors for each cell.

This results in an adjacency matrix *A*, where:

(1) $$A_{ij}=\left\{\begin{array}{cc} 1, & \mathrm{if}\;\mathrm{cell}\;j\;\mathrm{is}\;\mathrm{among}\;\mathrm{the}\;\mathrm{k-nearest}\;\mathrm{neighbors}\;\mathrm{of}\;\mathrm{cell}\;i\\ 0, & \mathrm{otherwise} \end{array}\right.$$

The adjacency matrix is then converted into an edge index representation, where each edge (i,j) corresponds to a nonzero entry in *A*. This sparse graph structure, implemented using kneighbors_graph(X, k) of the sklearn package [41], enables efficient processing in GNNs by capturing cell–cell relationships based on their chromatin accessibility-derived embeddings.

### 2.3. Extract Cell Embedding from Peak-by-Cell Matrix

Since the Peak-by-Cell Matrix encodes information about the chromatin accessibility of each cell across different genomic regions (peaks), it inherently exhibits high dimensionality, sparsity, noise, and batch effects between the reference and query datasets. These characteristics pose challenges for accurate cell-type annotation and require dimensionality reduction and batch effect correction to ensure robust downstream analysis. We first conducted feature selection, retaining the 300,000 most informative features to ensure that downstream analysis focused on biologically relevant chromatin accessibility signals. This step reduces noise while preserving key regulatory elements associated with cell identity. To further reduce dimensionality, we applied the spectral embedding method from SnapATAC2, downscaling the feature space to 32 dimensions. This technique efficiently captures the underlying manifold structure of the high-dimensional scATAC-seq data, ensuring that essential biological relationships are maintained.

To alleviate batch effects between the reference dataset and query dataset while preserving biological variability across tissue types, we performed mutual nearest neighbor (MNN) [42] batch correction. Specifically, we utilized the “mnc_correct” function in SnapATAC2, designating sample identity as the batch variable and tissue type as the grouping factor. This approach aligns shared cell populations across different datasets while retaining cell-type-specific variations.

### 2.4. Integrate DNA Sequence Information with Chromatin Accessibility Signals to Perform Cell-Type Annotation

To enhance the accuracy of scATAC-seq cell-type annotation, we leverage graph-based learning by integrating DNA sequence-derived embeddings with chromatin accessibility features. This approach transforms cell-type annotation into a node classification problem within a cell–cell graph. Specifically, the learned cell embeddings and constructed adjacency matrix above are input into a GAT, where each cell is treated as a graph node. The GAT model performs node classification, leveraging attention-based message passing to propagate labels from annotated reference cells to unlabeled query cells:

(2) $$h_{i}^{\prime }=\sigma \left(\sum_{j\in N(i)}\alpha_{ij}Wh_{j}\right)$$

where $h_{i}^{\prime }$ is the updated cell representation, *W* is the learned transformation matrix, $\alpha_{ij}$ represents the learned attention weight between cell *i* and its neighbor *j*, and N(i) denotes the neighborhood of cell *i*. Taken together, by combining sequence-derived adjacency information and chromatin accessibility features, the GAT model efficiently captures both sequence-intrinsic regulatory patterns and broader chromatin accessibility landscapes. This integration significantly enhances cell-type annotation accuracy, allowing for the precise identification of cell populations in complex single-cell ATAC-seq datasets.

#### Network Architecture and Training Details

The proposed architecture comprises two stacked GAT convolutional layers (GATConv), designed to facilitate effective information propagation across cells. The first layer employs a multi-head (4 heads) attention mechanism to selectively aggregate information from neighboring cells, enhancing the representation of chromatin accessibility patterns. This is followed by an exponential linear unit (ELU) [43] activation function, which ensures smooth feature transformations.The second GAT layer further refines the learned representations using a single attention head, which prioritizes the most relevant features for cell-type classification. A log-softmax activation function is then applied to produce probabilistic cell-type predictions, allowing for robust classification.

To optimize the model’s performance, training is conducted using a cross-entropy loss function, ensuring accurate cell-type assignments. Additionally, a binary cross-entropy loss is incorporated to facilitate domain adaptation, effectively mitigating distributional discrepancies between different datasets.The model is trained using the Adam optimizer [44] with a learning rate of 0.0001, and parameters are updated iteratively via backpropagation. During each training epoch, the model processes the input features and adjacency relationships, computes the classification loss on the labeled training set, and adjusts the parameters accordingly to improve predictive accuracy and generalization across datasets.

### 2.5. Benchmark Methods

To comprehensively evaluate the performance of scAttG in cell-type annotation, we selected three intra-omics methods specifically designed for annotating scATAC-seq data using scATAC-seq reference datasets, namely scAttG [45], Cellcano, scATAnno [9], and scNym [46]. Additionally, we included scJoint, which traditionally employs scRNA-seq as the reference dataset for cell-type annotation. However, in this study, we adapted scJoint by using scATAC-seq as the reference dataset, while retaining its original network architecture and loss function. This modification allows for a direct comparison of scAttG against both specialized intra-omics methods and an adapted cross-omics approach, ensuring a thorough benchmarking of its performance.

### 2.6. Benchmark Datasets

We obtained the raw scATAC-seq matrix data directly from the corresponding online repositories and binarized the matrices following established protocols in previous studies.

- WholeBrainA, WholeBrainB, Cerebellum, and PreFrontalCortex: These datasets were derived from the adult mouse brain atlas, accessible via the GEO accession number **GSE111586** or through the project website: http://atlas.gs.washington.edu/mouse-atac/data/, accessed on 14 August 2018. The data were generated using sciATAC-seq technology and aligned to the mm9 reference genome.
- MosA1_v1, MosA1_v2, MosA1_v3, MosA1_v4, MosA2, MosM1, MosM2, MosP1, and MosP2: These datasets represent anterior, middle, and posterior regions of the secondary motor cortex in the mouse brain and are available under GEO accession number **GSE126724**. They were generated using snATAC-seq technology and annotated based on the GRCm38 reference genome.
- PBMC_atlas and PBMC_10K: These human-derived datasets consist of peripheral blood mononuclear cells (PBMCs) profiled using scATAC-seq. Both datasets share the same set of 344,492 peaks and are commonly used for benchmarking cell-type annotation algorithms. PBMC_atlas contains 394,441 cells annotated into 14 cell types and serves as a comprehensive reference, while PBMC_10K includes 10,412 cells and is typically used as a query set for evaluating model generalization performance. It can be downloaded from the work in [9].

For more detailed information about the benchmark dataset, please refer to Table 1.

#### Evaluation Metrics

To evaluate the performance of cell-type annotation, we adopt two widely used classification metrics: Accuracy and Macro-F1 score.

Accuracy measures the proportion of correctly predicted labels over all samples and is defined as:

(3) $$\mathrm{Accuracy}=\frac{1}{N}\sum_{i=1}^{N}I\left(y_{i}={\hat{y}}_{i}\right)$$

where *N* is the total number of cells, $y_{i}$ is the true label of the *i*-th cell, ${\hat{y}}_{i}$ is the predicted label, and I(·) is the indicator function that returns 1 if the condition is true and 0 otherwise.

Macro-F1 score is the unweighted average of the F1 scores computed independently for each class. It is particularly effective for evaluating performance on imbalanced datasets:

(4) $$\mathrm{Macro}-F1=\frac{1}{C}\sum_{c=1}^{C}{F1}_{c}$$

Each class-specific F1 score is calculated as:

(5) $${F1}_{c}=\frac{2\cdot {\mathrm{Precision}}_{c}\cdot {\mathrm{Recall}}_{c}}{{\mathrm{Precision}}_{c}+{\mathrm{Recall}}_{c}}$$

where precision and recall for class *c* are given by:

(6) $${\mathrm{Precision}}_{c}=\frac{TP_{c}}{TP_{c}+FP_{c}},\;{\mathrm{Recall}}_{c}=\frac{TP_{c}}{TP_{c}+FN_{c}}$$

Here, $TP_{c}$, $FP_{c}$, and $FN_{c}$ denote the numbers of true positives, false positives, and false negatives for class *c*, respectively. Macro-F1 treats all classes equally, regardless of their support, and is thus a reliable metric for assessing performance across both major and minor cell types.

## 3. Results

### 3.1. Performance Evaluation Across Intra-Dataset Experiments

To evaluate the performance across intra-dataset experiments of the benchmark methods, each dataset was randomly divided into three subsets: training (70%), validation (10%), and testing (20%). A stratified sampling strategy was employed to ensure that the distribution of cell types remained consistent across all subsets. The training set was used to optimize the model parameters, the validation set was utilized for hyperparameter tuning and early stopping, and the query set was held out to assess final model performance. This evaluation protocol enables a rigorous assessment of the model’s generalization capability on data from the same distribution while minimizing the risk of overfitting.

**Figure 2:**
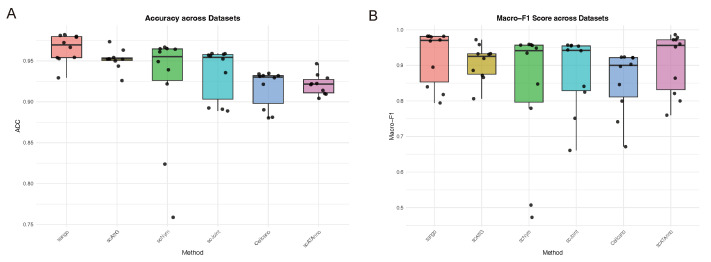
Performance evaluation across intra-dataset experiments. (**A**) Accuracy; (**B**) Macro-F1 score.

As shown in Figure 2, most methods demonstrate high levels of accuracy and Macro-F1 scores under this intra-dataset setting, which is largely attributed to the consistent distribution of cell types and the absence of significant batch effects. This homogeneity simplifies the classification task, thereby enhancing model performance. Among the evaluated approaches, scAttG and the proposed scAttG rank among the top performers across both metrics.

Although scAttG exhibits slightly lower accuracy than scAttG, it achieves superior robustness in terms of Macro-F1 score. This indicates that scAttG not only accurately classifies major cell populations but also excels in identifying minority cell types, reflecting its ability to handle class imbalance effectively. In contrast, alternative methods such as scNym and scJoint exhibit greater variability in performance across datasets, particularly with respect to Macro-F1, suggesting reduced stability in recognizing less prevalent cell types. These findings highlight the adaptability of scAttG to diverse intra-dataset structures and its enhanced generalization capacity. Consequently, scAttG offers a stable and reliable solution for cell-type annotation within homogeneous datasets and holds strong potential for practical deployment.

### 3.2. Performance Evaluation Across Datasets

**Figure 3:**
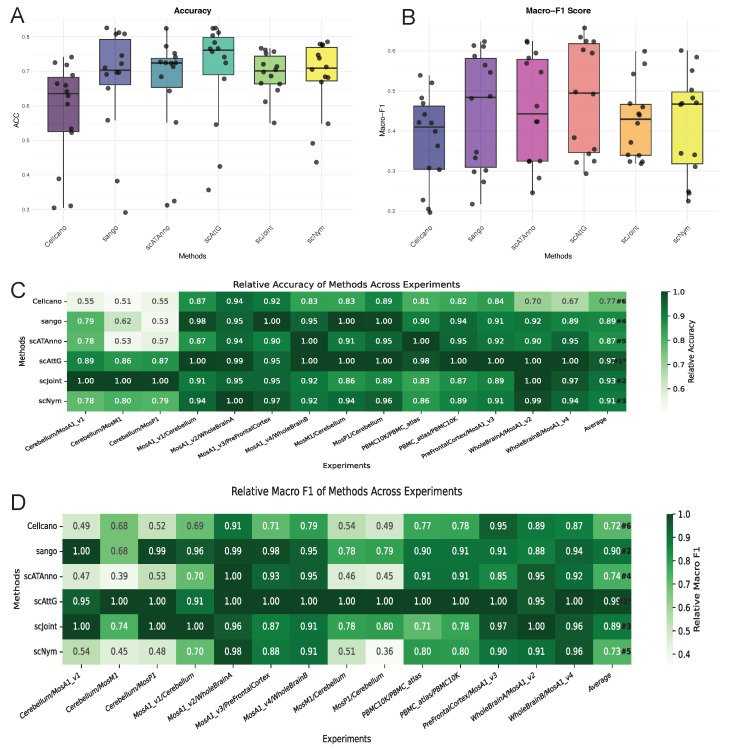
Comparison of scAttG with other methods. (**A**) Accuracy (box plot); (**B**) Macro-F1 score (box plot); (**C**) Relative accuracy; (**D**) Relative macro-F1 score.

In Section 3.1 above, the assessment was conducted under conditions without batch effects. However, in real-world scenarios involving cell-type annotation of scATAC-seq data, the reference and query datasets often originate from different tissues, experimental conditions, or sequencing platforms. These cross-dataset differences introduce substantial variability, making cell-type annotation significantly more challenging. To assess the robustness and generalizability of scAttG under such conditions, we conducted a series of cross-dataset experiments, as described in the following section. Specifically, the combinations of benchmark datasets used in the experiments are depicted along the *x*-axis of Figure 3C, where the dataset preceding the “/” is designated as the reference dataset, and the dataset following the “/” serves as the query dataset.

In the task of cell-type annotation for scATAC-seq data, the proposed method scAttG demonstrates notable advantages across multiple evaluation metrics. Firstly, regarding Accuracy (Figure 3A), scAttG achieves the highest median accuracy (about 0.75) among all compared methods. Moreover, the relatively narrow interquartile range indicates that its predictions are both stable and robust. In comparison, Cellcano and scJoint show lower median accuracies of approximately 0.65 and 0.68, respectively, with greater variability, suggesting less consistent performance. Secondly, in terms of the Macro-F1 score (Figure 3B), a metric particularly important for imbalanced datasets, scAttG attains a median value of approximately 0.44. This performance exceeds that of scAttG, Cellcano, scJoint, and scNym. The compact interquartile distribution further highlights the stability of scAttG and effectiveness in recognizing rare cell types. While scAttG demonstrates comparable upper-bound performance (reaching above 0.60 in some cases), scAttG exhibits superior consistency across experimental conditions. In summary, scAttG not only delivers high overall accuracy but also demonstrates robust performance in the face of class imbalance, a common challenge in scATAC-seq data. Its ability to maintain balanced classification across diverse cell types highlights its strong generalization capability and practical utility in real-world single-cell epigenomic analyses.

As illustrated in the heatmaps in Figure 3C and Figure 3D, the proposed scAttG method demonstrates remarkable stability and competitive performance across multiple experiments. In terms of relative accuracy, scAttG consistently achieves or approaches the optimal score in all 12 experimental settings, with an average value of 0.97, the highest among all compared methods. This indicates that scAttG performs reliably close to the best-performing method across diverse experimental conditions. Furthermore, in terms of relative Macro-F1 score, scAttG also ranks first, with an impressive average of 0.99, underscoring its particular strength in handling class imbalance. These results suggest that scAttG not only excels in overall accuracy but also maintains superior stability and inter-class balance, demonstrating strong generalization capability. Collectively, these findings establish scAttG as a highly robust and effective cell-type annotation method in complex single-cell analysis scenarios.

**Figure 4:**
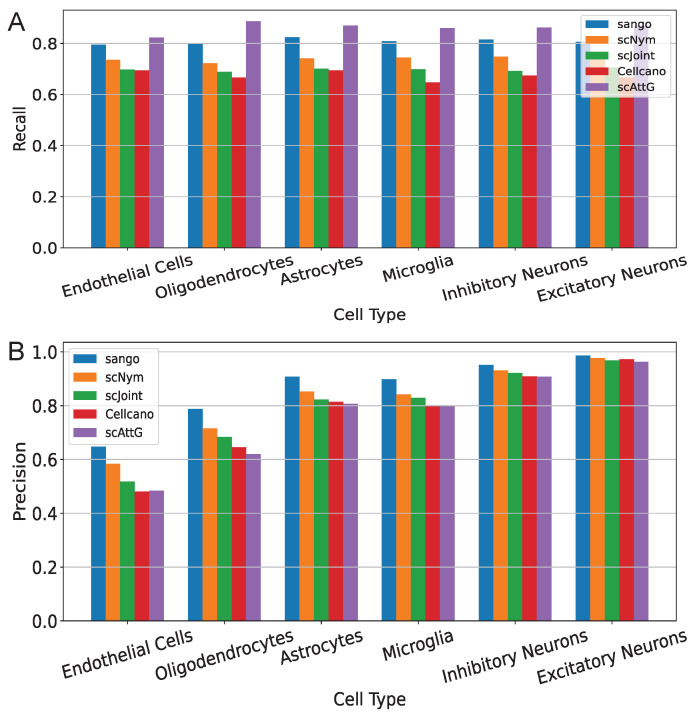
Comparison of per-cell-type performance across six annotation methods on the MosA1_v2/WholeBrainA experiment. (**A**) Recall; (**B**) Precision.

In addition, we evaluated the recall and precision of each method for individual cell types using the MosA1_v2/WholeBrainA experiment as a representative case (Figure 4). The results show that scAttG maintains stable performance across both abundant and rare cell types, highlighting its robustness to distributional shifts. Compared to baseline methods, scAttG achieves higher recall in region-specific neuronal subtypes and enhanced precision in glial cell populations, underscoring its adaptability and effectiveness in complex cell-type annotation scenarios.

### 3.3. Sankey Plot Analysis

**Figure 5:**
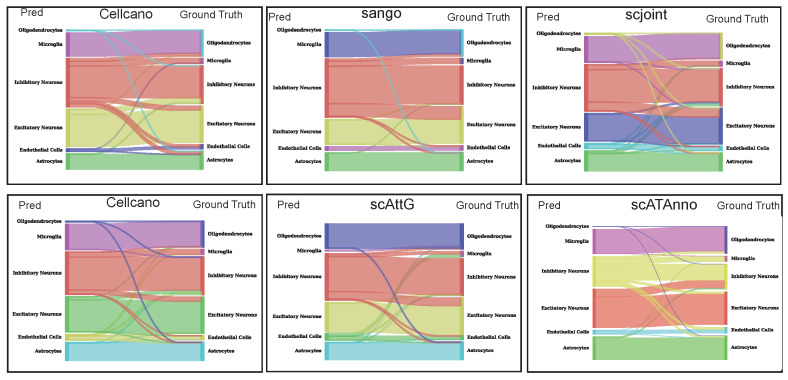
Sankey plot generated from the MosA1_v2/WholeBrainA experiment.

In the previous section, we evaluated the performance of scAttG at a macro level. Here, we focus on a representative experiment (MosA1_v2/WholeBrainA) to provide a more detailed assessment. As illustrated by the Sankey plot (Figure 5), scAttG demonstrates a clear advantage in the cell-type annotation task. Its predicted labels show high concordance with the ground truth, with major cell types such as Inhibitory Neurons, Excitatory Neurons, and Astrocytes exhibiting well-aligned and concentrated flows, with minimal misclassification. Notably, scAttG also accurately identifies rare cell types such as Endothelial Cells and Microglia, highlighting its robustness and generalization capability in handling both class imbalance and subtle inter-type differences. Overall, scAttG delivers superior cell-type annotation quality, reinforcing its state-of-the-art performance in scATAC-seq data analysis.

### 3.4. Two-Dimensional Embedding Analysis

**Figure 6:**
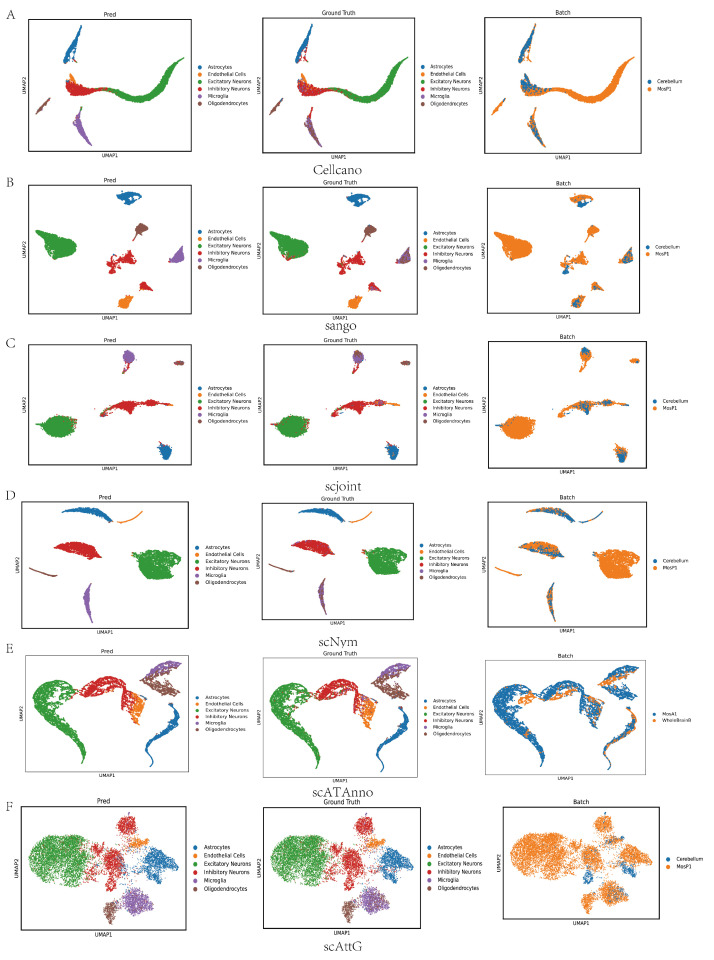
UMAP plots show scAttG achieves clear cell-type separation and effective batch correction across datasets. (**A**) Cellcano; (**B**) scAttG; (**C**) scjoint; (**D**) scNym; (**E**) scATAnno; (**F**) scAttG.

To further assess the performance of scAttG, we employed UMAP (uniform manifold approximation and projection) [47] for dimensionality reduction and visualization of the cell embeddings. The UMAP plot (Figure 6) illustrates the clustering of cells based on their predicted labels, providing insights into the model’s ability to separate different cell types in a two-dimensional space. As shown in the UMAP visualization in Figure 6, all methods demonstrate reasonably good performance in cell-type annotation; however, scAttG exhibits superior results. Its predicted labels align closely with the true annotations, with distinct and well-separated clusters for all cell types, indicating minimal confusion or misclassification. In the corresponding batch plot, cells of the same type from different batches are accurately co-located, demonstrating that scAttG effectively alleviates batch effects without overcorrection, thereby preserving the biological identity of each cell type. Compared to other methods, scAttG achieves a better balance between cross-sample consistency and cell-type resolution, highlighting its robustness and strong generalization capability in complex single-cell datasets.

### 3.5. Ablation Study

#### 3.5.1. Comparative Evaluation of Full and Ablated scAttG Models Accessibility Features

**Figure 7:**
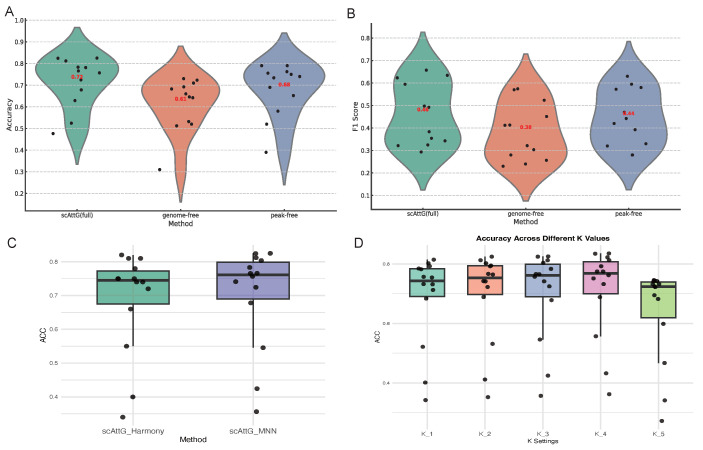
Evaluation of the impact of architectural and training choices on the performance of scAttG. (**A**,**B**) Violin plots showing accuracy (**A**) and macro-F1 score (**B**) for the full scAttG model compared to two ablated variants, genome-free and peak-free, across twelve benchmark datasets. The full model demonstrates superior consistency and performance in both metrics. (**C**) Comparison of batch correction strategies (Harmony vs. MNN) integrated within scAttG, with accuracy as the evaluation metric. (**D**) Accuracy across five different K values used in the graph construction step.

Figure 7 presents the comparative performance of the proposed scAttG (full) model and its two ablated variants across twelve datasets, evaluated using Accuracy and Macro–F1 score. The results are visualized using violin plots, where each black circle represents the score of an individual experiment and the red numeric labels indicate the mean performance for each method. Notably, scAttG (full) consistently outperforms the two ablation variants in both evaluation metrics, achieving an average Accuracy of approximately 0.73 and a Macro–F1 score of about 0.47, demonstrating both superior and stable performance across diverse conditions. The genome-free variant, which excludes genomic sequence information and constructs the neighbor graph solely from reduced peak features, exhibits a marked decline in performance. Similarly, the peak-free variant, which relies solely on CNN-derived genomic sequence features while omitting peak-based information, also fails to match the full model’s effectiveness. These findings underscore the value of integrating both genomic sequence features and chromatin accessibility peak structure, affirming the complementary nature of the two modalities in capturing cellular heterogeneity and enhancing the accuracy of cell-type classification.

#### 3.5.2. Comparison of Batch Correction Strategies: MNN vs. Harmony in scAttG

To evaluate the impact of batch correction strategies on the performance of *scAttG*, we compare MNN and Harmony across 14 cross-dataset experiments, as shown in Figure 7C. The figure illustrates the accuracy distributions for both variants, where each black dot represents the accuracy from a single experiment. The results demonstrate that *scAttG* integrated with MNN slightly outperforms the Harmony-based variant in terms of both median and overall accuracy. This suggests that MNN may be more effective than Harmony in preserving cell-type discriminability during batch correction for scATAC-seq cell type annotation.

#### 3.5.3. Sensitivity Analysis of KNN in scAttG

To assess the sensitivity of scAttG to the choice of K in the construction of the KNN graph, we conducted ablation experiments across five different K values (denoted as K_1 to K_5). Figure 7D presents the resulting accuracy distributions over 14 cross-dataset tasks. Each black dot represents the accuracy of a single experiment. The results show that, while overall accuracy remains relatively stable across different K values, values between K_2 and K_4 consistently yield slightly higher median performance. This suggests that moderate values of K offer a better tradeoff between local neighborhood sensitivity and global structure representation. Extremely small or large K values (e.g., K_1 and K_5) introduce greater variance and slightly reduced performance, indicating the importance of tuning K to maintain robust and discriminative graph construction.

## 4. Discussion

In this study, we present scAttG, a novel deep learning framework designed to enhance cell-type annotation for single-cell ATAC-seq data by integrating chromatin accessibility profiles and genomic sequence features. Through extensive benchmarking across twelve diverse scATAC-seq datasets, we demonstrate that scAttG achieves superior performance in both Accuracy and Macro-F1 score, surpassing state-of-the-art intra-omics methods.

One of the key advantages of scAttG lies in its dual-modality design, which leverages CNNs to capture DNA sequence-level features and GATs to propagate cell-type labels based on chromatin accessibility-derived neighbor relationships. This integration allows the model to exploit complementary information (regulatory motifs encoded in the DNA sequence and epigenomic patterns captured by chromatin accessibility), resulting in more robust and accurate cell representations. Our ablation study further underscores this point: removing either the sequence-based or peak-based module leads to significant performance degradation, confirming the synergistic effect of combining both modalities.

In real-world applications, batch effects caused by differences in experimental protocols, sequencing platforms, and tissue sources remain a significant obstacle to reliable cell annotation. scAttG addresses this challenge through a batch-aware design that corrects batch-associated peak reachability during feature learning and alignment. As evidenced by the cross-dataset experiments and UMAP visualization results, scAttG maintains high accuracy while effectively minimizing batch-induced variance. Notably, our model avoids overcorrection, preserving biologically meaningful variations across tissues and conditions.

Compared to existing intra-omics methods such as Cellcano, scNym, and scAttG, scAttG exhibits not only higher predictive accuracy but also greater consistency across datasets, as reflected in the compact interquartile ranges and high relative performance scores. Additionally, the model’s ability to annotate rare or underrepresented cell types (highlighted in Sankey plot analyses) demonstrates its efficacy in handling class imbalance, a prevalent issue in single-cell datasets.

Despite its promising performance, scAttG also presents several opportunities for future improvement. First, the current model relies on manually selected hyperparameters (e.g., number of neighbors k, CNN architecture depth), which may be further optimized using automated search strategies. Second, scAttG assumes that the reference annotations used for training are accurate; however, in real-world datasets, cell-type labels can be noisy or ambiguous. Incorporating mechanisms to handle label noise, such as robust loss functions, label smoothing, or noise-aware training paradigms, may improve model generalization and reliability, especially in large-scale or poorly annotated datasets. Third, extending scAttG to incorporate additional omics layers, such as DNA methylation or 3D chromatin structure, may further enhance cell identity resolution.

In summary, scAttG represents a significant advancement in the field of single-cell epigenomics, offering a principled and practical approach for accurate and stable cell-type annotation. By effectively integrating genomic and epigenomic signals, scAttG contributes a powerful tool for unraveling cellular heterogeneity and understanding regulatory mechanisms in complex biological systems.

## 5. Conclusions

We proposed scAttG, a novel deep learning framework that integrates DNA sequence features and chromatin accessibility data to enable accurate and robust cell-type annotation in scATAC-seq data. By combining CNNs for extracting sequence-level regulatory signals and GATs for modeling accessibility-derived cellular relationships, scAttG leverages complementary information to enhance cell representation learning. Through extensive benchmarking on a range of datasets, scAttG generally performs favorably compared to existing state-of-the-art methods, achieving competitive results in both annotation accuracy and stability. Its dual-modality architecture, along with a batch-aware learning strategy, ensures reliable generalization across different experimental conditions and tissue sources. Overall, scAttG provides a powerful and practical tool to advance the analysis of cellular heterogeneity and regulatory landscapes in single-cell epigenomic research.

## Figures and Tables

**Table 1:** Summary of scATAC-seq datasets used in this study.

Dataset	Tissue	#Peaks	#Cell Types	#Cells	GEO Accession
MosP1	nervous	68,555	6	10,889	GSE126724
Cerebellum	Cerebellum	68,555	16	2,278	GSE111586
MosA1_v1	nervous	67,300	6	11,034	GSE126724
MosM1	nervous	67,637	6	10,294	GSE126724
MosA1_v2	nervous	68,576	6	11,034	GSE126724
WholeBrainA	WholeBrain	68,576	17	5,494	GSE111586
MosA1_v3	nervous	68,578	6	11,034	GSE126724
PreFrontalCortex	PreFrontalCortex	68,578	18	5,959	GSE111586
MosA1_v4	nervous	68,567	6	11,034	GSE126724
WholeBrainB	WholeBrain	68,567	16	3,272	GSE111586
PBMC_atlas	PBMC	344492	14	394441	-
PBMC_10K	PBMC	344492	14	10412	-

## Data Availability

All datasets used in this study are publicly available and described in detail in the Section 2.6 section. The source code for our proposed method, including data preprocessing, CNN embedding, and GAT-based classification, is openly accessible on GitHub at: https://github.com/Guowei-nju/scAttG accessed on 1 May 2025.

## References

[1] Li H., Sun Y., Hong H., Huang X., Tao H., Huang Q., Wang L., Xu K., Gan J., Chen H. (2022). Inferring transcription factor regulatory networks from single-cell ATAC-seq data based on graph neural networks. Nat. Mach. Intell..

[2] Laisné M., Lupien M., Vallot C. (2025). Epigenomic heterogeneity as a source of tumour evolution. Nat. Rev. Cancer.

[3] Lu C., Wei Y., Abbas M., Agula H., Wang E., Meng Z., Zhang R. (2024). Application of Single-Cell Assay for Transposase-Accessible Chromatin with High Throughput Sequencing in Plant Science: Advances, Technical Challenges, and Prospects. Int. J. Mol. Sci..

[4] Jin W., Ma J., Rong L., Huang S., Li T., Jin G., Zhou Z. (2025). Semi-automated IT-scATAC-seq profiles cell-specific chromatin accessibility in differentiation and peripheral blood populations. Nat. Commun..

[5] Wang Y., Sun X., Zhao H. (2022). Benchmarking automated cell type annotation tools for single-cell ATAC-seq data. Front. Genet..

[6] Chen H., Lareau C., Andreani T., Vinyard M.E., Garcia S.P., Clement K., Andrade-Navarro M.A., Buenrostro J.D., Pinello L. (2019). Assessment of computational methods for the analysis of single-cell ATAC-seq data. Genome Biol..

[7] Ma W., Lu J., Wu H. (2023). Cellcano: Supervised cell type identification for single cell ATAC-seq data. Nat. Commun..

[8] Popescu M.C., Balas V.E., Perescu-Popescu L., Mastorakis N. (2009). Multilayer perceptron and neural networks. WSEAS Trans. Circuits Syst..

[9] Jiang Y., Hu Z., Lynch A.W., Jiang J., Zhu A., Zeng Z., Zhang Y., Wu G., Xie Y., Li R. (2023). scATAnno: Automated cell type annotation for single-cell ATAC sequencing data. bioRxiv.

[10] Zhang K., Zemke N.R., Armand E.J., Ren B. (2024). A fast, scalable and versatile tool for analysis of single-cell omics data. Nat. Methods.

[11] Korsunsky I., Millard N., Fan J., Slowikowski K., Zhang F., Wei K., Baglaenko Y., Brenner M., Loh P.r., Raychaudhuri S. (2019). Fast, sensitive and accurate integration of single-cell data with Harmony. Nat. Methods.

[12] Taunk K., De S., Verma S., Swetapadma A. A brief review of nearest neighbor algorithm for learning and classification. Proceedings of the 2019 IEEE International Conference on Intelligent Computing and Control Systems (ICCS).

[13] LeRoy N.J., Smith J.P., Zheng G., Rymuza J., Gharavi E., Brown D.E., Zhang A., Sheffield N.C. (2024). Fast clustering and cell-type annotation of scATAC data using pre-trained embeddings. NAR Genom. Bioinform..

[14] Chen X., Chen S., Song S., Gao Z., Hou L., Zhang X., Lv H., Jiang R. (2022). Cell type annotation of single-cell chromatin accessibility data via supervised Bayesian embedding. Nat. Mach. Intell..

[15] Goan E., Fookes C. (2020). Bayesian neural networks: An introduction and survey. Case Studies in Applied Bayesian Data Science: CIRM Jean-Morlet Chair, Fall 2018.

[16] Wang C., Krafft P., Mahadevan S., Ma Y., Fu Y. (2011). Manifold alignment. Manifold Learn. Theory Appl..

[17] Granja J.M., Corces M.R., Pierce S.E., Bagdatli S.T., Choudhry H., Chang H.Y., Greenleaf W.J. (2021). ArchR is a scalable software package for integrative single-cell chromatin accessibility analysis. Nat. Genet..

[18] Welch J.D., Hartemink A.J., Prins J.F. (2017). MATCHER: Manifold alignment reveals correspondence between single cell transcriptome and epigenome dynamics. Genome Biol..

[19] Stark S.G., Ficek J., Locatello F., Bonilla X., Chevrier S., Singer F., Rätsch G., Lehmann K.V. (2020). SCIM: Universal single-cell matching with unpaired feature sets. Bioinformatics.

[20] Liu J., Huang Y., Singh R., Vert J.P., Noble W.S. Jointly embedding multiple single-cell omics measurements. Proceedings of the Algorithms in Bioinformatics: The 19th International Workshop, WABI 2019 Proceedings, WABI (Workshop).

[21] Cao Z.J., Gao G. (2022). Multi-omics single-cell data integration and regulatory inference with graph-linked embedding. Nat. Biotechnol..

[22] Lowd D., Meek C. Adversarial learning. Proceedings of the Eleventh ACM SIGKDD International Conference on Knowledge Discovery in Data Mining.

[23] Dziugaite G.K., Roy D.M., Ghahramani Z. (2015). Training generative neural networks via maximum mean discrepancy optimization. arXiv.

[24] Scarselli F., Gori M., Tsoi A.C., Hagenbuchner M., Monfardini G. (2008). The graph neural network model. IEEE Trans. Neural Netw..

[25] Lin Y., Wu T.Y., Wan S., Yang J.Y., Wong W.H., Wang Y.R. (2022). scJoint integrates atlas-scale single-cell RNA-seq and ATAC-seq data with transfer learning. Nat. Biotechnol..

[26] Zhang Z., Yang C., Zhang X. (2022). scDART: Integrating unmatched scRNA-seq and scATAC-seq data and learning cross-modality relationship simultaneously. Genome Biol..

[27] Tian L., Xie Y., Xie Z., Tian J., Tian W. (2023). AtacAnnoR: A reference-based annotation tool for single cell ATAC-seq data. Briefings Bioinform..

[28] Yan X., Zheng R., Chen J., Li M. (2023). scNCL: Transferring labels from scRNA-seq to scATAC-seq data with neighborhood contrastive regularization. Bioinformatics.

[29] Khosla P., Teterwak P., Wang C., Sarna A., Tian Y., Isola P., Maschinot A., Liu C., Krishnan D. (2020). Supervised contrastive learning. Adv. Neural Inf. Process. Syst..

[30] Wang X., Qi G.J. (2022). Contrastive learning with stronger augmentations. IEEE Trans. Pattern Anal. Mach. Intell..

[31] Yigit H. A weighting approach for KNN classifier. Proceedings of the 2013 IEEE International Conference on Electronics, Computer and Computation (ICECCO).

[32] De Rop F.V., Hulselmans G., Flerin C., Soler-Vila P., Rafels A., Christiaens V., Gonzalez-Blas C.B., Marchese D., Caratu G., Poovathingal S. (2024). Systematic benchmarking of single-cell ATAC-sequencing protocols. Nat. Biotechnol..

[33] Scherer A. (2009). Batch Effects and Noise in Microarray Experiments: Sources and Solutions.

[34] Yuan H., Kelley D.R. (2022). scBasset: Sequence-based modeling of single-cell ATAC-seq using convolutional neural networks. Nat. Methods.

[35] Veličković P., Cucurull G., Casanova A., Romero A., Lio P., Bengio Y. (2017). Graph attention networks. arXiv.

[36] Li Z., Liu F., Yang W., Peng S., Zhou J. (2021). A survey of convolutional neural networks: Analysis, applications, and prospects. IEEE Trans. Neural Netw. Learn. Syst..

[37] Xiao S., Wang S., Dai Y., Guo W. (2022). Graph neural networks in node classification: Survey and evaluation. Mach. Vis. Appl..

[38] Tian C., Zheng M., Jiao T., Zuo W., Zhang Y., Lin C.W. (2024). A self-supervised CNN for image watermark removal. IEEE Trans. Circuits Syst. Video Technol..

[39] Yin W., Kann K., Yu M., Schütze H. (2017). Comparative study of CNN and RNN for natural language processing. arXiv.

[40] Sasse A., Ng B., Spiro A.E., Tasaki S., Bennett D.A., Gaiteri C., De Jager P.L., Chikina M., Mostafavi S. (2023). Benchmarking of deep neural networks for predicting personal gene expression from DNA sequence highlights shortcomings. Nat. Genet..

[41] Pedregosa F., Varoquaux G., Gramfort A., Michel V., Thirion B., Grisel O., Blondel M., Prettenhofer P., Weiss R., Dubourg V. (2011). Scikit-learn: Machine learning in Python. J. Mach. Learn. Res..

[42] Haghverdi L., Lun A.T., Morgan M.D., Marioni J.C. (2018). Batch effects in single-cell RNA-sequencing data are corrected by matching mutual nearest neighbors. Nat. Biotechnol..

[43] Clevert D.A., Unterthiner T., Hochreiter S. (2015). Fast and accurate deep network learning by exponential linear units (elus). arXiv.

[44] Zhang Z. Improved adam optimizer for deep neural networks. Proceedings of the 2018 IEEE/ACM 26th International Symposium on Quality of Service (IWQoS).

[45] Zeng Y., Luo M., Shangguan N., Shi P., Feng J., Xu J., Chen K., Lu Y., Yu W., Yang Y. (2024). Deciphering cell types by integrating scATAC-seq data with genome sequences. Nat. Comput. Sci..

[46] Kimmel J.C., Kelley D.R. (2021). Semisupervised adversarial neural networks for single-cell classification. Genome Res..

[47] McInnes L., Healy J., Melville J. (2018). Umap: Uniform manifold approximation and projection for dimension reduction. arXiv.

